# The effects of bilingualism on hippocampal volume in ageing bilinguals

**DOI:** 10.1007/s00429-021-02436-z

**Published:** 2022-01-05

**Authors:** Toms Voits, Holly Robson, Jason Rothman, Christos Pliatsikas

**Affiliations:** 1 Department of Language and Culture, UiT the Arctic University of Norway, Hansine Hansens veg 18, 9019 Tromsø, Norway; 2 Department of Psychology and Language Sciences, University College London, Chandler House, 2 Wakefield Street, London, WC1N 1PF UK; 3 Centro de Investigación Nebrija en Cognición, Universidad Nebrija, Calle de Sta. Cruz de Marcenado, 27, 28015 Madrid, Spain; 4 School of Psychology and Clinical Language Sciences, University of Reading, Harry Pitt Building, Earley Gate, Whiteknights Road, Reading, RG6 6AL UK

**Keywords:** Bilingualism, Ageing, Memory, Hippocampus, Structural MRI, Experience-based neuroplasticity

## Abstract

Long-term management of more than one language has been argued to contribute to changes in brain and cognition. This has been particularly well documented in older age, where bilingualism has been linked to protective effects against neurocognitive decline. Since memory difficulties are key aspects of this decline, herein we examine potential effects of bilingualism on the hippocampus, a brain structure related to memory that is particularly vulnerable to cognitive ageing. Hippocampal volume has been shown to increase as a result of second language learning and use in younger adults. However, it is unknown if this is maintained throughout the lifespan. We examine hippocampal volume and episodic memory performance in a participant sample consisting of healthy older individuals with a wide range of experiences in exposure and using a second language. Results reveal greater hippocampal volume calibrated to degree of quantified dual language use. Our results mirror those of immersive active bilingualism in younger populations, suggesting that long-term active bilingualism leads to neuroprotective effects in the hippocampus. We discuss this in the context of literature proposing bilingualism-induced brain reserve in the older age.

**Supplementary Information:**

The online version contains supplementary material available at 10.1007/s00429-021-02436-z.

## Introduction

Bilingualism has been shown to be a lifestyle enrichment factor correlating with greater neural plasticity across the lifespan, at least under conditions of active and sustained engagement (see Pliatsikas 2020 for review). Directly or indirectly, these outcomes are hypothesised to be a consequence of increased demands for executive and language control needed to manage more than one linguistic system in a single mind/brain (e.g., Grundy et al. 2017). Moreover, research has shown that ageing bilinguals outperform monolinguals in various domains of executive functioning, such as mental set shifting, updating, and inhibition (e.g., Bialystok et al. 2004; Sullivan et al. 2016, although see Lehtonen et al. 2018). With regard to neuroanatomy in older age, bilingualism has been associated with greater grey matter volume and white matter integrity across brain structures involved in bilingual language control, language learning, and language processing (Anderson et al. 2018a, b; Duncan et al. 2018; Gold et al. 2013). Such findings are of particular importance, as older age is the period in life where cognition and the brain naturally decline.

Typical cognitive ageing is most clearly identifiable in anatomical changes such as reductions in grey matter (GM) volume and/or white matter (WM) integrity, especially in the prefrontal cortex and the hippocampus, and/or decreased neural efficiency (i.e., increased recruitment of implicated networks) in task performance (e.g., Rönnlund et al. 2005; Persson et al. 2006; Giorgio et al. 2010; Nyberg et al. 2010; Bettio et al. 2017; Farokhian et al. 2017). However, there is a general variability in cognitive ageing trajectories across the population (see Cabeza et al. 2018 for review). Some individuals seem to be more resilient to age-related cognitive decline. In addition to genetic factors as determinants of individual differences, variability can be explained by the widely used concepts of cognitive and brain reserve (Stern et al. 2018). Cognitive reserve refers to preserved cognitive ability in the face of neural damage or atrophy, manifesting as better-than-expected cognition in cases of progressive neurodegeneration (Stern 2002). Brain reserve refers to the build-up of neural tissue, as a structural reinforcement of the brain, via volumetric increases caused by neurogenesis or dendritic branching (Valenzuela and Sachdev 2006). In individuals with increased brain reserve, neural decline may take longer before any cognitive and behavioural symptoms manifest.

Brain reserve is typically observed in healthy individuals and linked to a variety of lifestyle enrichment factors, such as higher education, physical exercise, demanding leisure activities, and high occupational attainment (Cabeza et al. 2018; Darwish et al. 2018; Foubert-Samier et al. 2012; Hötting and Röder 2013; Perneczky et al. 2019; Ritchie et al. 2019; Yaffe et al. 2009). Bilingualism also stands out as a potential lifestyle factor for reserve accrual. This is so because the mechanisms implicated in the mental stimulation/exercise required to efficiently maintain, manage, and use multiple languages overlap with those believed to be at the core of accrual for other lifestyle-enrichment factors. Indeed, while some studies report null results (Mukadam et al. 2017; Yeung et al. 2014; Zahodne et al. 2014), an increasing number of studies provide evidence of bilingualism contributing to the delay of dementia symptom onset in neurodegenerative diseases, most commonly in Alzheimer’s disease or Mild Cognitive Impairment (MCI) (e.g., Alladi et al. 2013; Bialystok et al. 2007; Calabria et al. 2020; see Anderson et al. 2020 for critical review).

Active bilingualism has been demonstrated to have implications for episodic memory performance in elderly adults (mean age 80 +) (Schroeder & Marian 2012). However, despite compelling reasons to the contrary, few studies have examined the hippocampus, a core element of the episodic memory network, in bilinguals at any age and none specifically in older bilinguals (DeLuca et al. 2019b; Mårtensson et al. 2012; Li et al. 2017). Since (active) bilingualism often correlates to later diagnosis of dementia (see Anderson et al. 2020 for critical review) and diagnosable symptoms often relate to real world memory issues, investigating brain areas potentially underlying this observation—structures and networks where memory is core—is timely and important.

**Fig. 1:**
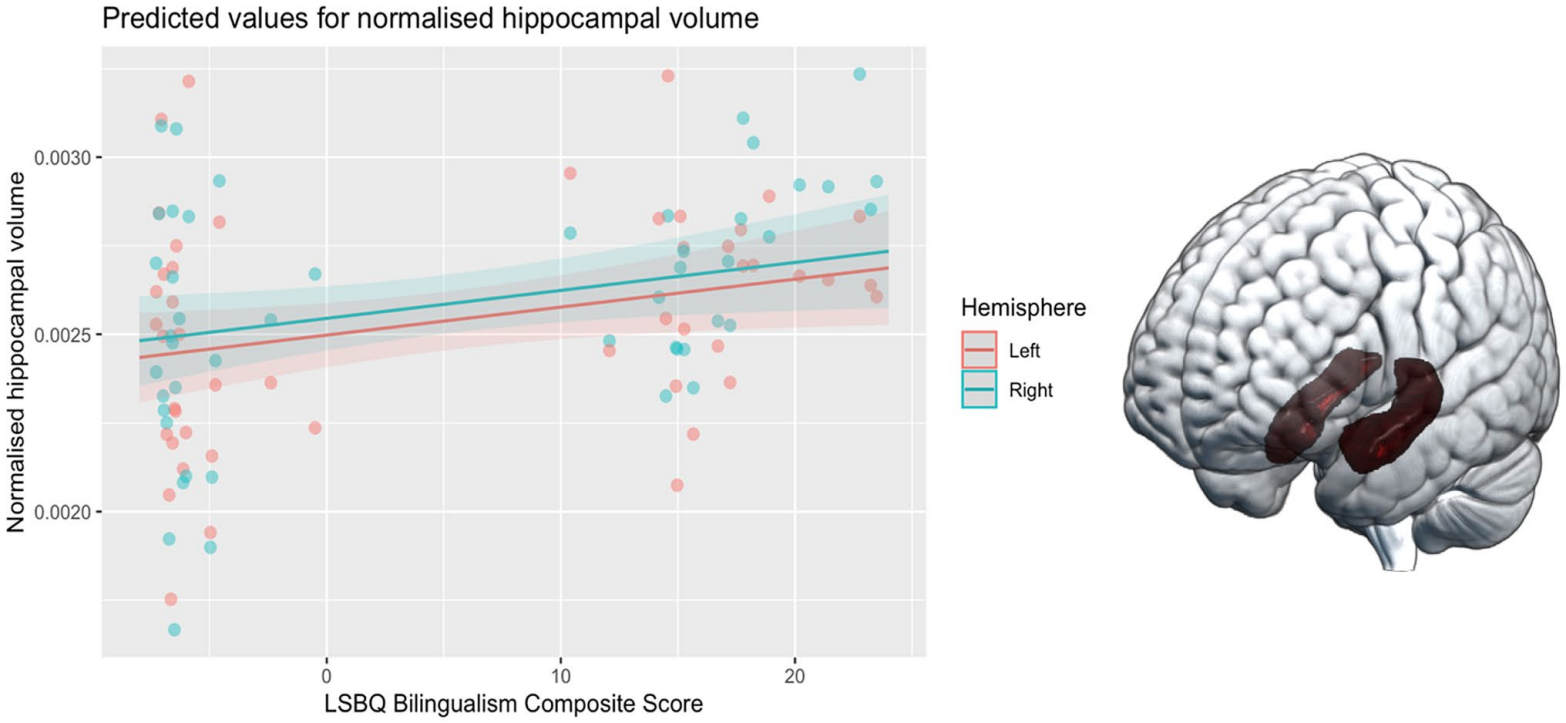
Plot of the predicted values of normalised hippocampal volumes against LSBQ BCS (Model 3), superimposed on observed data points. See Table 3 for statistical comparisons. Bilateral hippocampus shown on the right-hand side on an MNI template for illustrative purposes

The hippocampus is a bilateral grey matter structure in the medial temporal lobe (see Fig. 1), associated with supporting episodic memory function, but it also underlies other important aspects of cognition, such as recognition, spatial processing, language learning, emotional behaviour, vocabulary acquisition, and mental imagery (Anand and Dhikav 2012; Bellmund et al. 2018; Bird and Burgess 2008; Breitenstein et al. 2005; Ullman 2004). Previous work has linked reductions in hippocampal size with verbal and non-verbal episodic memory performance decline (Gorbach et al. 2017; O’Shea et al. 2016). The hippocampal anatomy is subject to decline in healthy ageing by annual volumetric loss of 0.79–2%, surpassing that of other brain structures (in comparison, annual gross brain volume reduces by 0.2–0.5%) and becomes increasingly rapid with older age (Fjell et al. 2009; Fraser et al. 2015). Moreover, hippocampal atrophy is an established indicator for conversion from healthy ageing to development of mild cognitive impairment (Fotuhi et al. 2012) and from latter to Alzheimer’s disease (Apostolova et al. 2006).

Notwithstanding, the hippocampus has been shown to be plastic in response to changes in behaviour. For example, behavioural or physical interventions can impact hippocampal volume and improve memory performance in the older age, effectively reversing age-related hippocampal tissue loss (Erickson et al. 2011; Firth et al. 2018; Lövdén et al. 2012). Early life intellectual enrichment has been linked to increased hippocampus volume (Sumowski et al. 2016), which in turn has been shown to contribute to cognitive resilience in the pathologically ageing brain (Erten-Lyons et al. 2009). In sum, not only does the hippocampus appear to be a malleable brain structure, but its malleability seems to have correlates with behaviour.

Not unlike other areas of the brain that bilingualism is argued to impact, data on how bilingualism affects the hippocampus are somewhat mixed, with most of the literature indicating hippocampal anatomy to be sensitive to bilingual language experience. Mårtensson et al. (2012) examined Swedish interpreter students and found a significant volumetric increase in the right hippocampus following an intensive 3-month language course. Bellander et al. (2016) also reported expansion of the right hippocampus in young Swedish speakers as they acquired Italian vocabulary over the course of 4 months. Interestingly, expansions were not related to the amount of vocabulary acquired, but to the amount of time spent studying the second language (L2), i.e., engagement with additional language learning overall. In a longitudinal study, DeLuca et al. (2019b) tested bilinguals living in an immersive L2 environment for 3 years, and reported significant reshaping of the right hippocampus in the form of simultaneous expansions and contractions of different subfields of the structure. Li et al. (2017) compared hippocampal volumes between highly proficient bimodal Mandarin Chinese–Chinese Sign Language bilinguals and Mandarin Chinese monolinguals (aged 29–67). They showed enlarged hippocampus for the former group, who reported active use of both their languages on a regular basis. In juxtaposition to the above, Olsen and colleagues (2015) did not find any volumetric differences in the hippocampus between 70-year-old bilinguals and monolinguals, although they did find differences in other parts of the temporal lobe.

One reason for some inconsistency in the findings might relate to how bilingualism itself is operationalised across studies [a more general issue in the cognitive neuroscience of bilingualism literature, see Leivada et al. (2021) for discussion]. Indeed, most studies looking at the effects of bilingualism on neurocognition have treated bilingualism as a discrete, binary variable (whereby one is categorised as monolingual or bilingual). Such an approach fails to acknowledge, much less capture, the dynamic nature of bilingualism and the ensuing potential variability across bilinguals. Recently, there has been a push to unpack individual differences *across* bilinguals. In this manner, bilingualism is treated in a more nuanced way, a *continuum,* by finding ways to qualify and quantify an individual’s bilingual experiences (Bak 2016; Bialystok 2017; De Cat et al. 2018; DeLuca et al. 2019a, b, 2020; Gullifer et al. 2018; Luk and Bialystok 2013). Beyond addressing the obvious question of whether or not bilingualism can result in neurocognitive adaptations observable in older age per se, one wants (if not needs) to isolate and better understand the conditions of language exposure/engagement that differentiate individual bilinguals along the trajectory of outcomes (Grundy 2020; Leivada et al. 2021).

### The present study

In the context of the above discussion, the present study focuses on individual differences of bilingual experience on the hippocampus in healthy ageing. Highly proficient speakers of English as a second language in long-term immersion and native English-speakers with ranging from limited to no working knowledge of other languages (functional monolinguals) underwent a behavioural and MRI testing battery assessing their memory and hippocampal structure. This was accompanied by a collection of detailed language background information permitting quantification of bilingual experience on an individual level along a spectrum. We treat language experience and regress it as a dynamic, continuous variable within a collapsed group of all participants (functional monolinguals and bilinguals) and then only within the self-identifying bilingual sample, as in Pliatsikas et al. (2021). In this way and in line with calls in the recent literature (Luk and Bialystok 2013; DeLuca et al. 2020; de Bruin 2019), we sidestep two potential comparative fallacies: (a) the assumption that monolinguals and bilinguals form a priori distinct groups and (b) that the members of either group are so similar to one another in relevant sense that that individual variation is trivial. In the case (a) or (b) is true or happens to apply to our sample, the collapsing we performed in our models that run proxies for *language engagement* would reveal this anyway.

We propose and test two hypotheses regarding bilingualism, hippocampal structure, and memory performance. In line with the previous findings in younger populations, we expected to see greater hippocampal volume predicted by increased bilingual exposure/engagement. Second, if language exposure/engagement contributes to increased hippocampal volume, we further predict better episodic memory performance correlated to increased hippocampal volume.

## Methods

### Participants

Forty-eight healthy older adults (30 females, *m*Age: 62.19, SD: 9.62, range 48–84) were recruited for the study. Of these, 23 self-identified as bilingual or multilingual speakers of L2 English (16 females, *m*Age: 58.48, SD = 6.77, range 49–73) (henceforth referred to as ‘bilinguals’) and 25 were functionally monolingual native English speakers, some of which had had some experience with other languages (e.g., second language learning at school age), but reported being able to hold a conversation only in English (mAge = 65.60, SD = 10.68, range 48–84) (henceforth referred to as ‘monolinguals’). All participants were right-handed and reported no neurological disorders or history of speech and language impairments, and they were all resident in the UK at the time of testing. Prior to participation, subjects provided written informed consent and reported no counterindications to MRI scanning. All participants scored within the normal range of the ACE-III (Hsieh et al. 2013), suggesting no indications of cognitive impairment. Behavioural testing and MRI scanning sessions were mostly conducted on the same day, although in some cases, where it was not feasible to conduct all aspects of testing in 1 day, participants returned for a second round of testing at a later date. The maximum time period between the testing sessions was 3 months.

The bilingual participants spoke a variety of first languages but converged on English being an additional language. Most of these participants (*N* = 22) reported speaking an additional language or languages to English and their respective L1. While this means that these individuals were not strictly bilinguals but brought different language backgrounds and experiences to the table, they all converged on the fact that they have a long-term engagement with bilingualism, while living immersed in an environment where their first language is not a majority one. This means that their bilingual language control processes are actively used, leading to potential changes in neurocognition. In terms of language proficiency, two bilingual individuals reported English to be their most proficient language, 16 reported English as their second most proficient language, three individuals reported English as their third most proficient language, and one reported English as their fourth most proficient language. These participants usually acquired English at school age (*m*AoA: 10.65; SD: 6.12; range 0–30). The majority of this group were born outside the UK and had moved to the UK at various ages. Two participants in this group were born in the UK, but did not speak English at home and started learning English upon commencement of formal education. One participant was born in the Netherlands and reported growing up in a bilingual Dutch/English household. Participants in this group had been immersed in their additional language environment for an extended period of time (mean length of residence in the UK = 29.52 years; SD = 17.20; range 1–60), and were using English for everyday communication and were competent and highly proficient users of this language (see Table 1). Of the self-reported monolinguals, 13 participants reported some exposure to an additional language, usually at school age. However, none of the monolinguals reported continuous engagement with their additional languages at the present day, mostly advising ‘occasional use while on holiday’. Active engagement with their L2 was normally in a classroom setting during adolescence, decades prior to testing.

**Table 1:** Demographic information

	Self-reported monolinguals (*n* = 25)	Self-reported bilinguals (*N* = 23)	Statistical comparison
Mean age (SD)	65.6 (10.7)	58.5 (6.77)	*p* = 0.008136
Sex	14 F; 11 M	16 F; 7 M	Chi-sq = 0.45078; *p* = 0.502
Education	19.1 (3.59)	20.5 (3.16)	*p* = 0.1569
LSBQ bilingualism composite score	− 5.94 (1.58)	17.0 (3.43)	*p* < 0.001
LSBQ L2 Home score	− 12.3 (2.50)	5.23 (4.52)	*p* < 0.001
LSBQ L2 Social score	− 6.06 (2.86)	51.8 (8.40)	*p* < 0.001
L2 English speaking proficiency (out of 10)	–	8.48 (1.28)	–
L2 English reading proficiency (out of 10)	–	8.95 (0.996)	–
L2 English writing proficiency (out of 10)	–	8.47 (1.37)	–
L2 English understanding proficiency (out of 10)	–	8.82 (1.16)	–
English age of acquisition (years; bilinguals only)	–	10.7 (6.12)	–
Length of L2 immersion (bilinguals only)	–	29.5 (17.2)	–

The Language and Social Background Questionnaire (LSBQ; Anderson et al. 2018a, b) (see Sect. Language and Social Background Questionnaire (LSBQ) for details) offers bilingualism composite scores under -3.12 as firmly bilingual and scores over 1.22 as firmly monolingual. Individuals scoring between these values lie in a ‘grey area’, with ambiguous language background. Although self-identifying as “monolinguals”, two participants scored between -3.12 and 1.22 in LSBQ; one of these participants had no working knowledge of any other languages but reported growing up in an environment where they were surrounded by other languages. The other participant reported extensive experience with French, although they were not actively engaging in use of French in their everyday life. This variation in scores alone shows the need to move past group comparisons and treat bilingualism as a more nuanced variable. No participants were excluded from analysis based on their linguistic background. For full language and demographic information, split by self-reported *‘-lingualism*’, see Table 1.

### Behavioural data collection

#### Language and Social Background Questionnaire (LSBQ)

The participants completed the language and social background questionnaire (LSBQ) (Anderson et al. 2018a, b). The LSBQ is a questionnaire that allows one to collect detailed information about one’s social (professional attainment, country of birth, etc.) and linguistic background (spoken languages, self-rated proficiency, age, and context of acquisition), and the extent of language use across different contexts (see Mann and de Bruin (2021), for recent work testing and highlighting the effectiveness of the LSBQ). Bilingual experience is quantified via a bilingualism composite score (BCS) as a sum of various quantitative experience-based factors such as extent of L2 use in home and social settings. The BCS allows for measurement and treatment of bilingualism as a continuous variable, as opposed to the more commonly used and now questioned stratification of participants in monolingual and bilingual language groups (see de Bruin 2019; Pliatsikas et al. 2020; Surrain and Luk 2019; Leivada et al. 2021).

The participants completed a paper copy of the LSBQ on their own, but an examiner was present to answer any questions participants may have had and provide clarification as needed. As LSBQ presumes English to be the native or first language by default, the calculations using the factor score calculator were canonical for the native English speakers, whereas the calculations for those with other first languages were altered for their native language to be treated as the baseline, and English regarded as L2. This required inversion of some scores from the questionnaire upon input in the factor score calculator (as in DeLuca, et al. 2019a, b).

#### NIH toolbox

A modified cognition battery of the NIH Toolbox (NIH-TB; Weintraub et al. 2013) was used to assess the cognitive functioning of the study participants. The NIH-TB is an iPad-based testing battery. For the present study, two tests were of particular interest: the NIH-TB Picture Sequence Memory Test (testing episodic memory performance) and NIH-TB List Sorting Test (testing working memory performance), discussed in detail below. Hippocampus is typically associated with episodic memory performance, while working memory relies on frontal and parietal networks (Nee and D’Esposito 2015). Nonetheless, hippocampal volume has been shown to correlate with performance in the specific NIH toolbox working memory task in ageing populations (O’Shea et al. 2016). Inclusion of two tasks tapping into different memory domains allows us to test for the specificity of the results to episodic memory function with the hippocampus and for involvement of this structure in working memory processes. In the List Sorting Working Memory Test, participants were presented with cartoon pictures of different foods and animals, with accompanying audio presentation and written text naming the item. The participants were then asked to repeat them back to the examiner listing them in size order from the smallest to the biggest. In the first condition, participants were asked to recall stimuli from one category. In the second condition, participants were presented with stimuli from two categories (foods and animals) in mixed order and required to recall the items in size order for each category separately. The number of items in each trial increases until two trials of the same length are failed. All items were of high frequency, easily recognisable, and unambiguous. The test is scored as the total items correct across all trials.

In the Picture Sequence Memory Test, sequences of pictured objects and activities were presented in a particular order. The participants were then asked to reproduce the same order on the screen. The pictures are presented in two trials: one with a 15-step sequence and the other with an 18-step sequence. The second sequence is a repetition of the same 15 items, with three novel items added in the middle of the sequence. The score is derived by the cumulative number of adjacent pairs remembered correctly over the learning trials.

Both NIH toolbox tests were automatically scored with uncorrected standard scores measuring behavioural performance. Moreover, age and education measures, also collected as part of the behavioural data via the NIH toolbox, were included in the analysis as covariates. The education scoring of the NIH toolbox takes into account the highest level of education achieved and estimates years of formal education from it.

#### Addenbrooke’s Cognitive Examination (ACE-III)

Participants were asked to complete the Addenbrooke’s Cognitive Examination (ACE-III) testing battery (Hsieh et al. 2013). ACE-III is a widely used screening tool for cognitive deficits in Alzheimer’s Disease and Frontotemporal Dementia. It is scored out of 100 and covers five cognitive domains—attention, memory, fluency, language, and visuospatial processing. An overall score of less than 82 is suggestive of potential dementia. The domain of primary interest in this study was memory. The tasks tapping into memory are scattered throughout the exam and tap into working, episodic, and semantic memory. More specifically, the participants are asked to recall previously repeated words, memorise and recall a fictional name and address, and recall well-known historically significant people (Bruno and Vignaga 2019). The memory domain is scored out of 26. The score provides a baseline information of one’s composite memory performance and was used in addition to the NIH toolbox cognitive battery episodic and working memory tasks. All participants performed within normal limits, indicating typical ageing (see Table 2).

**Table 2:** Neurocognitive measures and outcomes

	Self-reported monolinguals (*n* = 25)	Self-reported bilinguals (*N* = 23)	Statistical comparison *P*
Mean ACE-III total score	94.0 (4.67)	94.5 (3.72)	0.6713
Mean ACE-III memory domain score	23.8 (2.57)	25.0 (1.36)	**0.04888**
NIH-TB Episodic memory score	96.2 (12.4)	103 (11.7)	0.07583
NIH-TB Working memory score	97.5 (10.5)	99.7 (6.99)	0.4097
Total normalised hippocampal volume (× 10^3^)	4.90 (0.669)	5.36 (0.445)	**0.006894**
Left normalised hippocampal volume (× 10^3^)	2.44 (0.350)	2.64 (0.254)	**0.02545**
Right normalised hippocampal volume (× 10^3^)	2.46 (0.373)	2.72 (0.246)	**0.006478**

The significance level for bold should be defined at the level of p < 0.05

### MRI data acquisition

High-resolution T1 anatomical scans were acquired using an MPRAGE sequence on a 3 T Siemens MAGNETOM Prisma_fit MRI scanner, with a 32-channel Head Matrix coil and Syngo software (256 sagittal slices, 0.7 mm slice thickness, in-plane resolution 250 × 250, acquisition matrix of 246 × 256 mm, 224 mm FoV, TR = 2400 ms, TE = 2.41 ms, inversion time = 1140 ms, flip angle = 8°). The scan lasted approximately 10 min.

### MRI data processing

#### Pre-processing

Structural neuroimaging data were pre-processed and analysed with software pipelines in FSL. All T1-weighted scans were then anatomically pre-processed using the fsl_anat pipeline in FSL 5.0.9 (Smith et al. 2004). This involves a standard use of various MRI processing tools including the brain extraction tool (BET) used for skull stripping the raw T1 images and bias field correction as part of the pipeline. Bias field-corrected T1 images were used for segmentation of the hippocampus. The brain extractions were manually checked for quality control. This revealed that five participants had unsatisfactory extractions, which was addressed by applying custom extraction parameters and rerunning BET until we yielded satisfactory skull-stripped brain extractions. Manual extractions were checked and approved by two raters (TV and CP).

#### Volume

Segmentation of the bilateral hippocampus was performed using FIRST, a toolbox of FSL. FIRST performs registration, segmentation based on Bayesian appearance, and boundary corrections to produce segmented subcortical structures (Patenaude et al. 2011). Hippocampal extractions were verified visually and were not deemed satisfactory for one participant. While all other segmentations were performed on bias-corrected full T1 images, for the unsatisfactory segmentation, the pipeline was run again on the brain-extracted image, which produced a satisfactory subcortical segmentation of the structure. Hippocampal raw volumes were calculated using the fslstats tool. Hippocampal volume was normalised by dividing it by total intracranial volume as estimated from the skull stripped image.

#### Shape

As part of the FIRST pipeline, vertex analysis was also performed on the bilateral hippocampus to establish if BCS is a predictor for changes in the hippocampal shape. The standard procedure was implemented in FIRST, by which each structure was linearly registered (using 6 degrees of freedom) to the sample-specific average surface and mapped in MNI space. Analysis was carried out using the Randomise pipeline in FSL, in which permutation-based non-parametric analysis with 10,000 permutations for each factor of interest testing was ran and corrected for multiple comparisons using threshold-free cluster enhancement (Smith and Nichols 2009). The correlational design matrix contained the factor of interest, BCS, and covariates of age and education. This procedure resulted in spatial maps showing local contractions and expansions of the structure (i.e., perpendicular displacement from the study-specific template average surface) of interest as a function of bilingualism.

The participant with unsatisfactory hippocampal segmentations from the complete T1 scan had to be excluded from shape analysis as it could not be included in the generation of the study-specific template of the hippocampus. Therefore, the study-specific template of the hippocampal vertices for the shape analysis was created without this participant. Note that the manual extraction of the hippocampal volume from the brain-extracted image was successful for this participant.

### Volumetric analysis

We investigated whether degree of bilingualism predicts hippocampal volume beyond other demographic factors and memory performance. To do so, we used fixed effects of demographic measures, individual test scores from the NIH toolbox and ACE-III and the LSBQ BCS, and random effects of participant to build and compare several hierarchical linear mixed-effects model in an increasing order of complexity.^1^

The FIRST tool, used to segment the structures of interest, provides two volumetric values—one for the left hippocampus, and one for the right. Mixed-effects models were run to evaluate hippocampal volume on either hemisphere. The initial model (Model 1) explains hippocampus volume as a function of age, education, hemisphere, and random effects of participant. The second model (Model 2) adds memory performance measures as independent variables to the model. The decision to include memory performance measures as predictors in this model (whereas, more typically, one would see brain measures as predictors for behaviour) was done to account for the individual variance in the hippocampal volume, shown to account for behavioural performance in other studies. In other words, like the demographic variables, memory performance is effectively acting as a predictor of no interest. The third model (Model 3) introduces our main predictor of interest, the LSBQ BCS.^2^ Akaike Information Criterion (AIC) was established for all models using the anova() function to determine the goodness of fit and choose the most appropriate model for the data.

### Behavioural analysis

Pertaining to the second hypothesis, we aimed to explore if bilingualism as a continuous variable predicts memory performance when other variables, including age, education, and total hippocampal volume, are accounted for. This included running separate sets of hierarchical models for all three memory performance measures—NIH toolbox episodic memory score, NIH toolbox working memory score, and ACE-III memory score. The models (Models 4–7) were built in a similar manner to the volumetric analysis models of the hippocampus (Models 1–3), with the difference of exclusion of a fixed effect of hemisphere and random intercepts of participant. For this analysis, hippocampal volumes were summed across hemispheres and the total hippocampal volume was used as a predictor for memory performance. For model numbering purposes, models testing episodic memory were numbered by adding the letter E, working memory models—letter W, and ACE-III memory composite—letter C.

In variants of Model 4, each memory measure as a dependent variable was predicted by age and education as independent variables. In the following step, variants of Model 5, total hippocampal volume was added to the list of independent variables. LSBQ BCS was added as an independent predictor in variants of Model 6. Finally, to test if bilingualism interacts with the hippocampal volume to impact memory in variants of Model 7, we explored the interaction of the hippocampal volume and BCS. This model was built by expanding model 6 with an age by bilingualism composite score interaction term.

All variants of models 4–7 were checked for assumptions for linear regressions using the gvlma package (Peña and Slate 2006). They were met for models testing episodic memory performance as the dependent variable. However, one or more assumptions were violated for models testing working memory and ACE-III memory domain score, suggesting that results of these regression models may not be accurate.

## Results

### Neuroimaging results

#### Volume

Hierarchical mixed-effects models were used to investigate whether the observed increased hippocampal volumes can be predicted by the amount of bilingual experience (see Table 3). Results from Model 1 revealed a trending negative effect of age, such that with increasing age, the observed hippocampus volumes became smaller, and a trending positive effect of education where higher educational attainment predicts higher hippocampal volume. No significant effects of hemisphere were observed.

**Table 3:** Hippocampal volume model comparison (*p < 0.05; **p < 0.01; ***p < 0.001)

Predictors	Model 1					Model 2					Model 3				
	Estimates	SE	Std. Beta	Statistic	*p*	Estimates	SE	Std. Beta	Statistic	*p*	Estimates	SE	Std. Beta	Statistic	*p*
Intercept	0.003***	0.000	− 0.071	6.143	**< 0.001**	0.003***	0.001	− 0.071	4.040	**< 0.001**	0.003***	0.001	− 0.071	4.429	**< 0.001**
Age	− 0.000	0.000	− 0.232	− 1.779	0.082	− 0.000*	0.000	− 0.326	− 2.453	**0.018**	− 0.000	0.000	− 0.239	− 1.782	0.082
Education	0.000	0.000	0.229	1.752	0.087	0.000*	0.000	0.299	2.322	**0.025**	0.000*	0.000	0.275	2.213	**0.033**
Hemisphere	0.000	0.000	0.143	1.293	0.202	0.000	0.000	0.143	1.293	0.202	0.000	0.000	0.143	1.293	0.202
ACE-III memory						0.000	0.000	0.213	1.715	0.094	0.000	0.000	0.136	1.090	0.282
NIH-TB episodic memory						− 0.000*	0.000	− 0.286	− 2.036	**0.048**	− 0.000*	0.000	− 0.302	− 2.233	**0.031**
NIH-TB working memory						− 0.000	0.000	0.118	− 0.944	0.351	− 0.000	0.000	− 0.127	− 1.056	0.297
LSBQ BCS											0.000*	0.000	0.282	2.131	**0.039**
Observations	96					96					96				
Marginal *R*^2^/Conditional *R*^2^	0.133/0.719					0.225/0.729					0.280/0.731				
AIC	− 1297.5					− 1299.1					− 1302.2				

The significance level for bold should be defined at the level of p < 0.05

Results from Model 2 revealed that age and education became significant contributors to the hippocampal volume, whereas hemisphere was not. Moreover, performance in the episodic memory task of the NIH toolbox correlated negatively with hippocampal volume, whereas performance in the working memory task of the NIH toolbox and overall composite memory performance were not significantly associated with hippocampal volume.

Finally, Model 3 revealed that, while the effects of education, and episodic memory performance remained significant, BCS also emerged as a unique contributor to the hippocampal volume, with higher BCS being positively associated with hippocampal volume (see Table 3). Adding BCS to the model increased the marginal *R*^2^ value from 0.225 to 0.28 and AIC decreased from − 1299.1 to − 1302.2 when comparing model 3 to model 2, indicating better explanatory power of the model by including BCS as a predictor. The lowest AIC indicating the best model fit for the data was for the most complex model (Model 3).

A version of model 3 (Model 3b) was also run on the bilingual subsample, to test the claim that higher amount of bilingual experiences correlates with change in neural anatomy. Within the bilingual participant group, the effect of LSBQ BCS was trending at *p* = 0.0514. This is not necessarily an unexpected outcome, as the power was drastically reduced, by including only 23 participants in this analysis, and LSBQ captures scores across the spectrum of *-lingualism*. No other predictor but episodic memory performance (*p* < 0.01) was significant in this version of the model (see Table 4).

**Table 4:** Model 3b (Model 3 on the bilingual subsample) (*p < 0.05; **p < 0.01; ***p < 0.001)

Predictors	Model 3b				
	Estimates	Std. Error	Std. Beta	Statistic	*p*
Intercept	0.003*	0.001	− 0.153	2.593	**0.020**
Age	− 0.000	0.000	− 0.255	− 1.120	0.279
Education	0.000	0.000	0.238	1.276	0.220
Hemisphere	0.000	0.000	0.306	1.617	0.120
ACE-III memory	0.000	0.000	0.099	0.554	0.588
NIH-TB episodic memory	− 0.000*	0.000	− 0.591	− 2.715	**0.015**
NIH-TB working memory	0.000	0.000	0.043	0.239	0.814
Bilingualism Composite Score	0.000	0.000	0.370	2.105	0.051
Observations	46				
Marginal *R*^2^/conditional *R*^2^	0.303/0.651				

The significance level for bold should be defined at the level of p < 0.05

To test whether bilingualism affects the hippocampus specifically, we ran a control model (Model 3c) with all predictors remaining as independent variables but substituting hippocampal volume with normalised brainstem volume as the dependent variable. Brainstem was chosen as a comparison variable to the hippocampus as there is no theoretical reason to believe that bilingualism has any effect on brainstem volume. The results showed that, unlike for the hippocampal volume, bilingualism did not significantly predict brainstem volume, indicative of specificity for bilingualism effects to hippocampal volume. Statistics for this model are reported in the Supplemental material (Table S1).

#### Shape

Vertex analysis revealed no significant local expansions or contractions of the bilateral hippocampus as a function of BCS, thresholded at *p* =  < 0.05.

### Behavioural results

As no effect of hemisphere emerged in the first set of models, we summed the left and right hippocampal volumes and used total hippocampal volume as a predictor in the sets of models testing behavioural performance across memory domains. For the NIH toolbox working memory task, hierarchical regressions showed that none of the predictors (age, education, total hippocampal volume, BCS, or BCS by hippocampal volume interaction) significantly explained working memory performance (see Models 4W, 5W, 6W, 7W, Table 5).

**Table 5:** Behavioural hierarchical regression

NIH toolbox working memory																				
Predictors	Model 4W					Model 5W					Model 6W					Model 7W				
	Estimates	Std. Error	Std. Beta	Statistic	*p*	Estimates	Std. Error	Std. Beta	Statistic	*p*	Estimates	Std. Error	Std. Beta	Statistic	*p*	Estimates	Std. Error	Std. Beta	Statistic	*p*
Intercept	96.019 ***	13.367	− 0.109	7.183	** < 0.001**	107.038 ***	18.556	− 0.085	5.768	** < 0.001**	107.818 ***	18.562	− 0.093	5.809	** < 0.001**	107.982 ***	18.743	− 0.126	− 0.619	** < 0.001**
Age	− 0.053	0.143	− 0.057	− 0.372	0.712	− 0.086	0.148	− 0.092	− 0.582	0.563	− 0.043	0.154	− 0.046	− 0.280	0.781	− 0.051	0.156	− 0.054	− 0.324	0.748
Sex	2.620	2.724	− 0.292	− 0.962	0.341	− 2.041	2.814	0.227	0.725	0.472	2.222	2.818	0.248	0.789	0.435	2.211	2.845	0.246	0.777	0.442
Education	0.245	0.402	0.093	0.608	0.546	0.345	0.420	0.132	0.821	0.416	0.325	0.420	0.124	0.773	0.444	0.349	0.427	0.133	0.816	0.419
Hippocampal volume						− 2092.507	2436.229	− 0.143	− 0.859	0.395	− 2833.634	2540.280	− 0.192	− 1.115	0.271	− 2903.647	2569.176	− 0.162	− 0.861	0.265
LSBQ BCS											0.131	0.128	0.173	1.023	0.312	− 0.428	1.233	0.144	0.790	0.730
Hippocampal volume × LSBC BCS																104.820	229.931	0.085	0.456	0.651
Observations	48					48					48					48				
*R*^2^/*R*^2^ adjusted	0.037/− 0.028					0.054/− 0.034					0.077/− 0.033					0.081/− 0.053				
AIC	354.067					355.251					356.068					357.825				

Performance in the NIH toolbox working memory task (*p < 0.05; **p < 0.01; ***p < 0.001)

The significance level for bold should be defined at the level of p < 0.05

For the NIH toolbox episodic memory task, age was a significant predictor in the Model 4E and remained a significant predictor in Models 5E, 6E, and 7E, so that with increased age, episodic memory performance is subject to decline. Education was a significant positive predictor in Models 5E, 6E, and 7E. Hippocampal volume also predicted episodic memory performance in Model 6E and 7E; however, the relationship was negative. In Model 6E, BCS did not significantly contribute to episodic memory performance. From all four episodic memory models, Model 6E was also the best fit for data with an adjusted *R*^2^ of 0.239 and the lowest AIC indicating the best fit (see Table 6).

**Table 6:** Behavioural hierarchical regression

NIH toolbox episodic memory																				
Predictors	Model 4E					Model 5E					Model 6E					Model 7E				
	Estimates	Std. Error	Std. Beta	Statistic	*p*	Estimates	Std. Error	Std. Beta	Statistic	*p*	Estimates	Std. Error	Std. Beta	Statistic	*p*	Estimates	Std. Error	Std. Beta	Statistic	*p*
Intercept	109.970***	16.733	0.012	6.572	** < 0.001**	140.677***	22.427	0.061	6.273	** < 0.001**	142.013***	22.150	0.052	6.411	** < 0.001**	141.655***	22.233	0.104	6.372	** < 0.001**
Age	− 0.435*	0.178	− 0.338	− 2.435	**0.019**	− 0.527**	0.179	− 0.410	− 2.944	**0.005**	− 0.453*	0.184	− 0.353	− 2.467	**0.018**	− 0.437*	0.185	− 0.340	− 2.357	**0.023**
Sex	− 0.402	3.410	− 0.033	− 0.118	0.907	− 2.016	3.401	− 0.163	− 0.593	0.556	− 1.705	3.363	− 0.138	− 0.507	0.615	− 1.681	3.375	− 0.136	− 0.498	0.621
Education	0.832	0.503	0.231	1.655	0.105	1.111*	0.507	0.308	2.191	**0.034**	1.077*	0.501	0.298	2.150	**0.037**	1.025*	0.507	0.284	2.023	**0.050**
Hippocampal volume						− 5831.010	2944.427	− 0.289	− 1.980	0.054	− 7100.063*	3031.373	− 0.352	− 2.342	**0.024**	− 6947.135*	3047.537	− 0.402	− 2.280	**0.028**
LSBQ BCS											0.224	0.152	0.215	1.469	0.149	1.445	1.462	0.261	0.988	0.329
Hippocampal volume × LSBC BCS																− 228.955	272.742	− 0.135	− 0.839	0.406
Observations	48					48					48					48				
*R*^2^/*R*^2^ adjusted	0.206/0.152					0.273/0.205					0.308/0.226					0.320/0.220				
AIC	375.628					373.439					373.035					374.217				

Performance in the NIH episodic memory task (*p < 0.05; **p < 0.01; ***p < 0.001)

The significance level for bold should be defined at the level of p < 0.05

For the ACE-III cognition battery memory domain, across all four models, no independent variables significantly predicted composite memory performance, apart from a significant main effect of sex in Models 4C and 5C, a trend for BCS in Model 6C (*p* = 0.078), suggesting that higher BCS might predict better performance in the ACE-III memory domain (see Table 7).

**Table 7:** Behavioural hierarchical regression

ACE-III composite memory score																				
Predictors	Model 4C					Model 5C					Model 6C					Model 7C				
	Estimates	Std. Error	Std. Beta	Statistic	*p*	Estimates	Std. Error	Std. Beta	Statistic	*p*	Estimates	Std. Error	Std. Beta	Statistic	*p*	Estimates	Std. Error	Std. Beta	Statistic	*p*
Intercept	24.612***	3.070	0.253	8.016	** < 0.001**	22.730***	4.279	0.236	5.312	** < 0.001**	23.044***	4.169	0.223	5.528	** < 0.001**	23.024***	4.217	0.240	5.460	** < 0.001**
Age	− 0.009	0.033	− 0.040	− 0.269	0.789	− 0.003	0.034	− 0.014	− 0.092	0.927	0.014	0.035	0.064	0.411	0.683	0.015	0.035	0.068	0.431	0.668
Sex	− 1.449 *	0.626	− 0.675	− 2.315	**0.025**	− 1.350 *	0.649	− 0.629	− 2.080	**0.044**	− 1.277	0.633	− 0.595	− 2.017	0.050	− 1.275	0.640	− 0.594	− 1.992	0.053
Education	0.041	0.092	0.066	0.445	0.659	0.024	0.097	0.038	0.248	0.806	0.016	0.094	0.025	0.169	0.867	0.013	0.096	0.021	0.134	0.894
Hippocampal volume						357.382	561.741	0.102	0.636	0.528	58.882	570.554	0.017	0.103	0.918	67.749	578.040	0.000	0.117	0.907
LSBQ BCS											0.053	0.029	0.291	1.835	0.074	0.123	0.277	0.307	0.445	0.659
Hippocampal volume × LSBC BCS																− 13.275	51.732	− 0.045	− 0.257	0.799
Observations	48					48					48					48				
*R*^2^/*R*^2^ adjusted	0.112/0.052					0.120/0.039					0.186/0.089					0.187/0.068				
AIC	212.852					214.4021					212.6991					214.622				

Performance in the ACE-III memory component (*p < 0.05; **p < 0.01; ***p < 0.001)

The significance level for bold should be defined at the level of p < 0.05

Unlike the models explaining the volumetric variation of the hippocampus as a result of demographic variables, memory performance, and bilingualism, the linear regression models explaining the variance in memory performance were not a good fit for the data. In all cases, model comparison revealed the increasingly complex models not to improve their explanatory power over the data. The only exception to this were models explaining NIH episodic memory scores as a function of the above-described IVs, where most complex model offered a marginal improvement over the simpler models (*p* = 0.064). Therefore, only the episodic memory performance can be measured as a function of age, education, hippocampal volume, and bilingualism. See hierarchical regression model comparison for all three memory scores in Tables 5, 6, 7.

No significant interaction of bilingualism and hippocampal volume was revealed on either memory measurement.

## Discussion

In the present study, we examined the effects bilingualism might have on the ageing brain with a particular focus on the hippocampus and related cognitive abilities. The present results align with the previous studies, showing that bilingualism can affect the volume of the hippocampus (Bellander et al. 2016; DeLuca et al. 2019b; Li et al. 2017; Mårtensson et al. 2012), extending them to older populations. Notably, through quantification of linguistic exposure/engagement and treatment of this factor as a continuum, data show that greater engagement in second language use predicts increased hippocampal volumes across individuals.

Going back to the two hypotheses offered in the outset of the paper, these results are confirmatory of the first one. Our findings are in line with the claim that continuous engagement with an additional language presents differential structural reinforcement of the brain (Borsa et al. 2018; Pliatsikas et al. 2017). Similar effects are not uncommon among studies looking at brain structure in bilingualism. The cognitively demanding experiences of acquiring and controlling two languages lead to structural adaptations of implicated areas resulting in increased efficiency (Abutalebi et al. 2012; Hayakawa and Marian 2019). Notably, such adaptations are dynamic in nature, with initial temporal tissue increases potentially being followed by return to baseline volume but with reinforced local connections (Pliatsikas 2020), which, in turn, could be more resistant to age-related decline. Based on this, our findings can have one of a few possible explanations. To start, the observed difference could simply reflect a volumetric increase with greater bilingual engagement *par excellence* prior to any onset of cognitive ageing, similar to what has been claimed for such findings in younger bilinguals.

Alternatively, if natural decline is already in the process of taking place, the correlation of larger hippocampal volume with bilingual engagement could actually signify one of two things. The first possibility is that decline of the hippocampus happens at a *slower rate* for the bilinguals, whether or not they started the process of decline with larger hippocampi. However, this cannot be readily assumed given evidence that volumetric increases can return to baseline (retraction) with increased, enduring efficiency over time (DeLuca et al. 2019b). Conversely, it is possible that decline happens at a similar rate across the participant sample, whereby the greater volume we capture in our temporal snapshot at the time of imaging is a remnant of the previous volumetric change that in fact did not return to baseline. Under either scenario, we have clear evidence that bilingualism boosts resilience against age-related deterioration of the hippocampus or, more generalisably, can provide a *brain reserve* (Stern et al. 2018).

The particular age range of our participants and the cross-sectional design of the study do not allow to differentiate between the above scenarios. Nevertheless, evidence from this exact pivotal point in cognitive ageing might prove useful in explicating effects of bilingualism later in life and/or under pathological neurodegeneration (Berkes et al. 2020; Costumero et al. 2020; Duncan et al. 2018). Most notably, our finding that individual-level engagement with bilingual experiences can affect the hippocampus structurally follows from similar findings in younger bilinguals (DeLuca et al. 2019b). Importantly, the present study constitutes the first piece of evidence that brain reserves specifically in older bilinguals are modulated by individual-level factors related to how one interacts with their languages. This alone is an important finding, because it clarifies the confines and parameters under which effects of bilingualism are likely to take place, reasonable to predict and worthy of serious consideration to be promoted as best practice for amelioration of age-related decline and neurodegeneration (e.g., Voits et al. 2020).

Hypothesis two related to effects bilingualism might have on the performance in cognitive domains typically associated with the hippocampus—most notably, episodic memory. It was hypothesised that if bilingualism had a measurable effect on hippocampal volume, positive behavioural effects would likely co-occur. Episodic and working memory performance in our samples was tested with three separate tasks. With the potential effects of demographic factors, such as age and educational attainment, as well as hippocampal volume all accounted for, bilingualism did not emerge as a significant predictor for memory performance across any of the tests we administered. Thus, hypothesis two was disconfirmed. In fact, an unexpected finding resulted: our data show a negative relationship between hippocampal volume and episodic memory performance. This is especially surprising given that positive associations of episodic memory and the hippocampus have been widely reported in adjacent literatures (Anand and Dhikav 2012; O’Shea et al. 2016). A potential explanation might stem from the fact that episodic memory performance is not uniquely reliant on hippocampal volume. Rather it is a network of cortical, subcortical, and medial temporal lobe structures that work in tandem (Dickerson and Eichenbaum 2010). Although the hippocampus plays a prominent role in this network, one might need to investigate the structural integrity of this network as a whole, which is beyond the scope of the present paper. In any case, a positive brain-to-behaviour relationship is intuitive, often empirically shown and theoretically reasonable. Thus, the general asymmetry we report is at first glance perplexing. We now turn to ponder how to best make sense of these juxtaposed outcomes.

Why the discrepancy between the effects of bilingualism on brain structure and cognitive performance? One explanation could be the average age of our participants being only 62 years. This puts them on the ‘younger’ end of the ageing spectrum. While some ageing processes may have already begun, these participants are still cognitively healthy with no signs of memory impairment, attested by the near-ceiling score in the ACE-III memory domain. Nevertheless, our structural findings indicate that the processes that underlie the building of a brain reserve are already in action, but without measurable equivalents in behaviour. This pattern is reminiscent of evidence, suggesting that the mapping of behaviour to brain *function* is not always straightforward, at least in healthy populations (Abutalebi et al. 2012; DeLuca et al. 2020).

If on the right track, our participants, whether bilingual or monolingual, are not deep in the process of cognitive ageing. As a result, the behavioural asymmetry evidence could help to specify which of the scenarios articulated above for the monolingual/bilingual difference in hippocampal volume is more likely to be on target. It would follow from this line of reasoning that there is a volumetric increase for bilinguals (on a sliding scale relative to linguistic experience), prior to any significant cognitive ageing effects. If so, this would ostensibly leave the brain-behaviour performance asymmetry perplexing only to the extent that a mapping between hippocampal volume and increased memory performance must follow. On second thought, however, we submit that the asymmetry is not overly surprising nor concerning. In our data, generalised memory task performance is at or near ceiling across the board, which alone may indicate the lack of significant cognitive ageing in our participants. Given the very high performance, it is reasonable to assume that the granularity of the memory tasks is simply not sufficient to capture potentially underlying differences in memory represented by increased volume in the bilinguals. When study participants perform at ceiling on a given behavioural task, it is functionally impossible to further test whether all individuals are equipotential for the construct of interest. In other words, we cannot preclude that bilinguals with increased exposure/engagement relative to others (monolinguals or bilinguals with less engagement) do not have better memory resources overall. Rather what we know for sure is that everyone has sufficient memory resources to perform these specific tasks at ceiling. The behavioural tasks used in this study can be viewed, then, as a limitation. Behavioural effects of bilingualism have been shown to manifest (or not) depending on task difficulty (Costa et al. 2009). And so, future studies should employ more difficult cognitive tasks where individuals would be less likely to score at ceiling.

An inspirational study for the present one was that of Schroeder and Marian (2012) in which a positive correlation between bilingualism and memory performance was shown. However, the bilinguals in that study had a mean age of 80 + . As age increases, the effects of cognitive ageing and neuropathology can escalate exponentially (Fox and Schott 2004). Taken together, it could be the case that our participants, while older, are not old enough as in Schroeder and Marian (2012) to exhibit behavioural differences in episodic memory, at least on the specific tasks we used. All things being equal, we might expect that the bilingual participants in Schroeder and Marian (2012) to have similar or even more signs of neural atrophy to the hippocampus despite greater behavioural performance compared to their controls. Why? Because in their age range, one would expect that accrued neural reserve is being or has been exhausted and the compensation processes for neural atrophy (behaviour task corollaries of cognitive reserve) play a principal role. This, of course, is an empirical question—and a future avenue of research—as the relevant data do not exist. To the extent that using (up) of neural reserve precedes, even if overlapping to some degree, the behavioural effects of cognitive reserve, the present data would add support to this argumentation. A clear example of this is a recent study where monolinguals and bilinguals were matched on brain health (unlike the more commonly used matching on cognitive performance), which showed a bilingualism-related maintenance of cognitive status at equal levels of brain decline in ageing individuals (Berkes et al. 2021). In sum, we interpret the observed volumetric brain evidence an index of brain reserve.

Finally, the volumetric changes across the participant sample did not translate into significant effects on hippocampal shape. This lacking relationship is more challenging to interpret, not least as it leaves some questions for understanding the relationship between volume and shape as they are assessed by our tools. Regardless, this does not seem to be unprecedented within the relevant literature where the few available studies have, similarly to our data, reported effects on one metric only (volume or shape), but not both (DeLuca et al. 2019b; Li et al. 2017; Mårtensson et al. 2012).

Our findings call for further and more focused investigations on bilingual engagement effects on the ageing brain, and in particular on age ranges similar to ours, where the first signs of cognitive decline might emerge. Of course, the ultimate goal in this programme is to reveal the exact mechanisms of how bilingual experience and increased executive control demands impact episodic memory; however, addressing this properly requires much more research and sits beyond the scope of this study. Longitudinal designs would enable further, and more precise examination of onset and trajectory of any relationship bilingualism has in exponents of cognitive ageing as well as their underlying mechanisms. Crucially, focused studies similar to this one are required with pathologically ageing populations too, to add to a small but growing literature that will help us better understand the potential clinical implications both in healthy and pathological ageing (Voits et al. 2020). Moreover, our results suggest that given the observed decoupling of brain structure and behaviour, augmenting studies of behavioural task performance with methodologies that directly look under the proverbial hood simultaneously are especially welcome to assess the effects of bilingualism on the brain.

## Supplementary Information

Below is the link to the electronic supplementary material.

Supplementary file1 (DOCX 16 KB)

## Data Availability

Data will be made available on Openneuro and/or OSF and accession numbers provided on request.

## Footnotes

1 Given the participant age, we did not use age of acquisition as a reliable predictor as many bilingual individuals reported learning English at school, which may not accurately portray the actual engagement in English use across the lifespan and at the time of testing.

2 A version of Model 3 was also envisaged with L2 home and L2 social use scores as measures of bilingualism, and the interaction of two, instead of the LSBQ composite score. However, as these scores heavily contribute to the LSBQ composite score (L2 Home and L2 Social scores have 33% and 30% weighting in the BCS calculation), and they were highly correlated between them (cor = 0.95; p < 0.001), it was deemed inappropriate to include these scores as separate predictors in the same model as they introduce multicollinearity issues.

## Code availability

Code will be made available on OSF.
