# Supplementary material for: The effects of bilingualism on hippocampal volume in ageing bilinguals
Source: Brain Struct Funct. 2022 Jan 5;227(3):979–94. doi: 10.1007/s00429-021-02436-z (PMC8930894; doi:10.1007/s00429-021-02436-z)
Supplement: Supplementary file 1 — Supplementary file1 (DOCX 16 KB) [file 429_2021_2436_MOESM1_ESM.docx]

# **Supplemental materials**

*Table S1. A version of model 3 with Brainstem as a control region (Model 3c).*

| **Brainstem volume** | | | | | |
| --- | --- | --- | --- | --- | --- |
|  | **Model 3c** | | | | |
| *Predictors* | *Estimates* | *std. Error* | *std. Beta* | *Statistic* | *p* |
| (Intercept) | 0.012 ^***^ | 0.003 | -0.000 | 3.562 | **0.001** |
| Age | -0.000 | 0.000 | -0.139 | -0.945 | 0.350 |
| Education | 0.000 ^***^ | 0.000 | 0.555 | 4.074 | **<0.001** |
| ACE-III memory | 0.000 | 0.000 | 0.211 | 1.539 | 0.132 |
| NIH-TB episodic memory | -0.000 | 0.000 | -0.286 | -1.924 | 0.061 |
| NIH-TB working memory | 0.000 | 0.000 | 0.047 | 0.359 | 0.721 |
| LSBQ BCS | -0.000 | 0.000 | -0.095 | -0.656 | 0.516 |
| Observations | 48 | | | | |
| R^2^ / R^2^ adjusted | 0.336 / 0.239 | | | | |
| ** p<0.05   ** p<0.01   *** p<0.001* | | | | | |
